# High-density Mapping Facilitates Successful Ablation of Postincisional Right Atrial Flutter After Previous Mechanical Mitral Valve Replacement

**DOI:** 10.19102/icrm.2021.120101S

**Published:** 2021-01-15

**Authors:** Bernhard Strohmer, Franz Danmayr, Johannes Kraus, Markus Lassnig, Uta C. Hoppe

**Affiliations:** ^1^Salzburger Landeskliniken, Paracelsus Private Medical University Salzburg, Salzburg, Austria

**Keywords:** Atypical atrial flutter, high-density mapping, postincisional atrial reentrant tachycardia

A 44-year-old woman presented with permanent atypical atrial flutter (AFL) with a constantly elevated heart rate of 115 bpm due to 2:1 ventricular conduction. The patient had a long-standing history of chronic polycystic kidney disease with three complicated transplantations resulting in chronic hemodialysis and multiple shunt revisions. After severe sepsis with endocarditis, she underwent mechanical mitral valve replacement (29 mm) seven years ago. Rhythm control was ineffective despite four attempted direct-current cardioversions and she was referred for catheter ablation to prevent tachymyopathy. Computed tomography imaging of both atrial chambers was used to enhance electroanatomical mapping with the EnSite™ system. Entrainment pacing excluded cavotricuspid isthmus–dependent right and perimitral left atrial flutter. Right atrial mapping with the Advisor™ HD Grid Mapping Catheter, Sensor Enabled™ (18,700 map points; 2,800 points used) revealed a reentrant circuit covering the complete cycle length of 270 ms **(Video 1)**. The activation map demonstrated a wavefront around the anterolateral superior vena cava involving the postincisional roofline toward the interatrial septum from previous valve surgery. Highly fractionated (150 ms), low-amplitude (0.1–0.3 mV) signals were recorded along the presumed atriotomy, delineating the early-meets-late region **(Figure 1)**.

Due to documented episodes of sinus and junctional bradycardia, ablation was performed after pace termination during sinus rhythm and phrenic nerve pacing for safety reasons. An irrigated contact-force ablation catheter was used (30 W) to target abnormal signals severing the upper reentry circuit. The linear lesion was extended in a superior to inferior direction, connecting adjacent scar borders (< 0.1 mV) according to the voltage map. Noncapture along the ablation line as well as noninducibility of AFL were considered as valid endpoints of the procedure. With the restoration of stable normal sinus rhythm (53–106 bpm), the patient improved clinically and showed no recurrences of atrial tachyarrhythmias during follow-up.

## Figures and Tables

**Figure 1:**
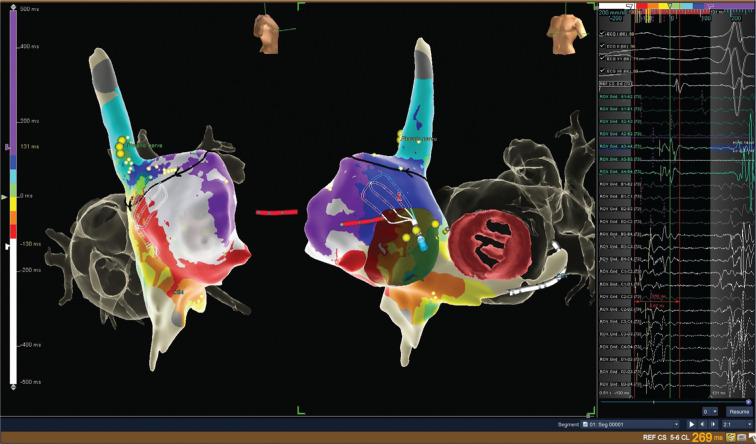
Activation mapping of the right atrial chamber was performed with the sensor-enabled Advisor™ HD grid catheter (right and left anterior oblique projections). The postincisional right atrial flutter (cycle length: 270 ms) demonstrated an upper reentrant circuit (depicted by the black arrow) involving previous atriotomy lines for mitral valve replacement. Highly fragmented, low-amplitude signals were detected (right panel) using the Advisor™ HD Grid catheter and served as a successful ablation target in the early-meets-late zone along the anterolateral superior vena cava/right atrial appendage region.

**Video 1.** Propagation mapping of the right atrial chamber (right and left anterior oblique projections) demonstrated postincisional right atrial flutter (cycle length: 270 ms) with an upper reentrant circuit (depicted by the black arrow) involving previous atriotomy lines for mitral valve replacement. The lower part of the right atrium was activated passively, and cavotricuspid isthmus–dependent flutter was excluded easily with entrainment pacing. Highly fragmented, low-amplitude signals (covering 150 ms) were detected with Advisor™ HD Grid catheter mapping (right panel) and served as a successful ablation target in the early meets late zone along the anterolateral superior vena cava/right atrial appendage region.

